# Inhibition mechanism of human galectin-7 by a novel galactose-benzylphosphate inhibitor

**DOI:** 10.1111/j.1742-4658.2011.08414.x

**Published:** 2012-01

**Authors:** Geoffrey Masuyer, Talat Jabeen, Christopher T Öberg, Hakon Leffler, Ulf J Nilsson, K Ravi Acharya

**Affiliations:** 1 Department of Biology and Biochemistry, University of BathUK; 2 Organic Chemistry, Lund UniversitySweden; 3 Section MIG, Department of Laboratory Medicine, Lund UniversitySweden

**Keywords:** drug design, galactoside inhibitor, galectin-7, lectin

## Abstract

Galectins are involved in many cellular processes due to their ability to bind carbohydrates. Understanding their functions has shown the necessity for potent and specific galectin inhibitors. Human galectin-7 (hGal-7), in particular, has been highlighted as an important marker in many types of cancer by either inhibiting or promoting tumour growth. Producing ligands able to selectively target hGal-7 will offer promising tools for deciphering cancer processes in which hGal-7 is involved as well as present potential solutions for future therapeutics. Here we report the high resolution crystal structure of hGal-7 in complex with a synthetic 2-*O*-benzylphosphate-galactoside inhibitor (which is > 60-fold more potent than its parent galactoside). The high resolution crystallographic analysis highlights the validity of using saccharide derivatives, conserving properties of the galactose binding, while enhanced affinity and specificity is provided by the added phosphate group. This structural information will allow the design of further inhibitors with improved potency and specificity.

**Database:**

The atomic coordinates for the complex of human galectin-7 as well as for the free structure have been deposited with the Protein Data Bank (accession numbers 3ZXE and 3ZXF respectively)

**Structured digital abstract:**

- hGal-7 and hGal-7 bind by X-raycrystallography (Viewinteraction)

## Introduction

Galectins are members of the carbohydrate-binding lectin family with specificity for β-galactosides. They have in common a carbohydrate recognition domain (CRD) and significant sequence similarity [1]. So far 15 mammalian galectins have been identified; they are classified in three subunit types based on their CRD architecture: prototype (galectin-1, -2, -5, -7, -10, -11, -13, -14, -15), tandem repeat type (galectin-4, -6, -8, -9, -12) and chimera-type (galectin-3) [2]. The CRD is about 130 amino acids long, and the β-galactoside binding site is conserved among galectins, while differences both in adjacent β-strands and loop regions explain the variation in oligosaccharide binding affinity [3,4].

These lectins are expressed by a wide range of cell types and can be found from the nucleus to the cytosol as well as being secreted in the extracellular space. They display various physiological roles in development, infection [5] and immunity [6] and have increasingly been linked with cancers [7]. The extent of galectin roles in these mechanisms is still unclear as they are involved in many cell–cell and cell–matrix interactions, as well as intracellular processes [8–10].

Understanding the role of galectins has raised the need for potent and selective inhibitors, which will be valuable tools for drug design in the treatment of galectin-mediated pathologies. The multivalence of galectins and the common CRD motif with different specificities towards particular carbohydrates are the keys to the function of these proteins. Several approaches have been successful in giving evidence towards the targeting of galectins for cancer treatment, such as the inhibition of metastasis with anti-galectin-3 monoclonal antibody in breast cancer cells [11]. The use of small molecules capable of directly binding the CRD seems like the most attractive option and has been demonstrated by specific synthetic peptides and carbohydrate-based inhibitors in malignant endothelial cells [12,13] and small molecule inhibitors in papillary thyroid cancer [14]. More recently galectin-1, a prototype galectin, was also identified as a target of choice for stopping cancer progression [15,16].

Human galectin-7 (hGal-7) is a 15 kDa prototype galectin with a single CRD, monomeric but capable of dimerization in solution [17]. It was first reported in an effort to identify markers of keranocyte differentiation [18]. Galectin-7 involvement in the maintenance of the pluristratified epithelia and epidermal stratification [19] has highlighted its role in wound healing. It was proven to be an efficient growth factor with therapeutic implications [20]. Some of the more recent advances on galectin-7 have shown its implication in apoptose induction in various types of cell. Galectin-7 expression is induced upon UV radiation [21] and regulated by p53, therefore showing high levels in certain types of cancer. Consequently galectin-7 has shown major roles in cancer development, by helping either in the elimination of certain tumour types [22] or in the growth stimulation of others [23,24]. Galectin-7 was recently described as a key element in aggressive metastasis following its overexpression in breast carcinomas and thus represents an interesting molecule as a marker for this pathology, and also as a therapeutic target [25].

The crystal structure of hGal-7 and its recognition of a range of carbohydrates have been described [26]. The crystal structure (PDB code 1BKZ) showed a dimeric arrangement allowing for the CRD presentation, which was confirmed by the structures of complexes with galactose, galactosamine, lactose and *N*-acetyllactosamine (PDB codes 2GAL, 3GAL, 4GAL and 5GAL respectively). The detailed map of hGal-7 binding to carbohydrates identified the key residues involved in the CRD and provided clues to the protein function as well as opening the way for current research into the design of small molecule inhibitors.

Salameh *et al.* [27] presented the synthesis of C3′-thioureido *N*-acetyllactosamine derivatives and Cumpstey *et al.* [28] that of substituted phenyl thio-β-d-galactopyranosides, both of which were potent inhibitors of galectin-7 with improved binding affinity to reported natural saccharides. Such designed ligands based on the galectin key affinity for galactose in combination with additional structural moieties that can interact with the CRD and surrounding unexploited sub-sites allows for optimization of affinity and specificity. Following such a design strategy, a series of 2- and 3-*O*-substituted galactosides **2**–**6** were recently synthesized and evaluated as inhibitors against a panel of galectins [29]. In this study, galectin-7 inhibition was enhanced by a 3-*C*-benzamido substitution of galactosides; cf. **1** and **2** (Table 1). Furthermore, addition of anionic 2-*O*-substituents to obtain a 2-*O*-(*H*-phosphonate) **3** and 2-*O*-alkylphosphate substituents (**5**, **6**) provided significantly enhanced binding to galectin-7, while a 2-*O*-phosphate **4** proved to enhance the affinity to a lesser extent for galectin-7. Together with phenyl thio-β-d-galactopyranosides [27] compounds **3** and **5**, **6** belong to the most potent monosaccharide inhibitors described for galectin-7. The three-dimensional structure of galectin-7 in complex with these newly designed inhibitors, such as the 2-*O*-benzylphosphate **6**, will help address the structural basis of the molecules’ inhibitory action, offer a platform for improving their specificity and become the template for efficient drug design. Here we report the improved high resolution crystal structure of hGal-7 at 1.4 Å, and its 1.7 Å structure in complex with the 2-*O*-benzylphosphate inhibitor **6**.

**Table 1:** Affinity of carbohydrate derivative inhibitors for hGal-7 (1–6 [29]; 7 [32]). *K*_d_ values are the average and standard deviation of repeated single point measurements at 4 °C

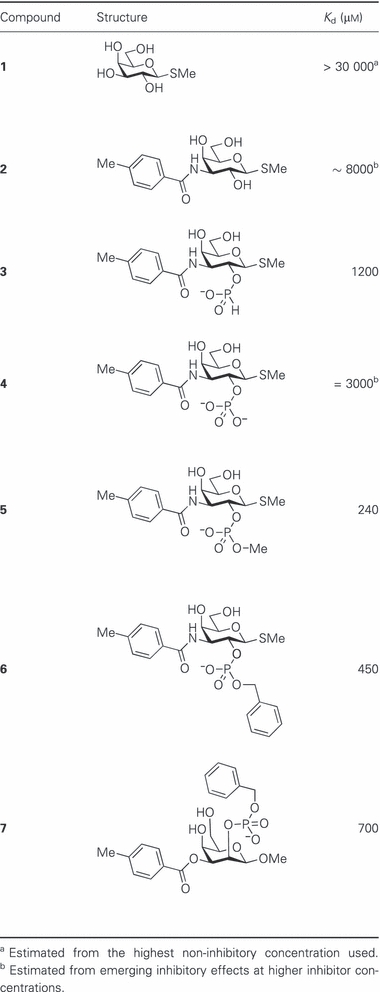

## Results

### Overall structure

**Fig. 1:**
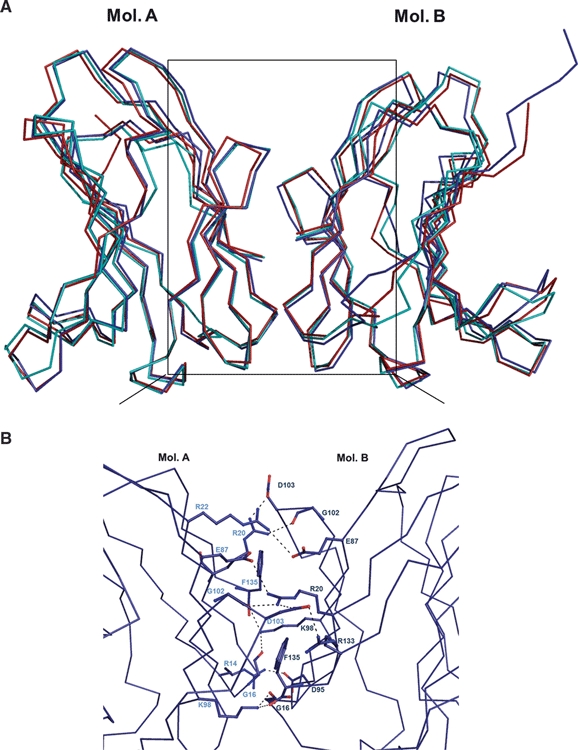
Structures of the high resolution hGal-7 and hGal-7 complex: previous hGal-7 structure (PDB code 1BKZ, red), hGal-7 high resolution structure (blue), and hGal-7 in complex with compound **6** (cyan, compound **6** not shown). Ribbon representation of the three superposed structures with molecule A and B of the dimer in the asymmetric unit.

The crystal structure of hGal-7 in complex with a novel inhibitor was determined at 1.7 Å, along with a higher resolution structure (1.4 Å) of the native form. In both cases, hGal-7 crystallized as a dimer in the P2_1_2_1_2_1_ space group, although the presence of the inhibitor changed the unit cell dimensions from *a* = 54.30, *b* = 65.11, *c* = 70.83 Å to *a* = 35.3, *b* = 53.5, *c* = 138.6 Å respectively (Table 2). The dimeric interface based on the β-strand (F1–F5 of both monomers) interaction is similar in both cases (Fig. 1). It was observed on SDS/PAGE and gel filtration chromatography that hGal-7 can form a dimer in solution as well as being present in its monomeric form (results not shown); others showed the dimer by analytical ultracentrifugation [17]. A pisa analysis [30] of the dimeric state in the crystal structure indicates that the interface area of 797 Å^2^ is potentially associated with 15 hydrogen bonds and 20 salt bridges; thus this interaction is probably due to crystallographic packing.

**Table 2:** X-ray data collection and refinement statistics

	hGal-7 native	hGal-7 + compound **6** complex
Data collection statistics		
Space group	P2_1_2_1_2_1_	P2_1_2_1_2_1_
Number of molecules/asymmetric unit	2	2
Cell dimensions	*a* = 54.30, *b* = 65.11, *c* = 70.83 Å; α = β = γ = 90°	*a* = 35.3, *b* = 53.5, *c* = 138.6 Å; α = β = γ = 90°
Resolution range (Å) (outer shell)	50–1.38 (1.42–1.38)	49.88–1.67 (1.71–1.67)
*R*_symm_^a^ (%)	6.2 (31.6)	6.6 (13.4)
*I*/*σI* (outer shell)	25.0 (3.5)	20.4 (9.6)
Completeness (outer shell), %	97.5 (95.2)	92.9 (84.4)
Redundancy (outer shell)	5.3 (4.3)	4.3 (4.5)
Total no. of reflections	561 283	248 037
Unique no. of reflections	52 544	31 402
Wilson *B* factor (Å^2^)	18.8	15.1
Refinement statistics		
Resolution range (Å)	47.9–1.4	69.3–1.7
*R*_cryst_^b^ (%)	18.1	20.4
*R*_free_^c^ (%)	22.6	23.9
Number of non-H atoms		
Protein	2193	2205
Ligand		33
Water molecules	336	376
Average temperature factor (*B* factor) (Å^2^)	20	17.6
rmsd in bond lengths (Å)	0.01	0.01
rmsd in bond angles (°)	1.26	1.08

a *R*_symm_ = Σ_*h*_Σ_*i*_|*I*(*h*) − *I*_*i*_(*h*)|/Σ_*h*_Σ_*i*_*I*_*i*_(*h*), where *I*_*i*_(*h*) and *I*(*h*) are the *i*th reflection and the mean measurements of the intensity of reflection *h*, respectively.

b *R*_cryst_ = Σ_*h*_|*F*_o_ − *F*_c_|/Σ_*h*_*F*_o_ where *F*_o_ and *F*_c_ are the observed and calculated structure factor amplitudes of reflection *h*, respectively.

c *R*_free_ is equal to *R*_cryst_ for a randomly selected subset of reflections not used in the refinement.

The high resolution (1.4 Å) structure gives an accurate insight into the molecular architecture of the protein with 99.3% of residues in the favoured Ramachandran plot area and 336 water molecules observed. The overall ‘jelly-roll’ fold is conserved compared with the previously obtained native and carbohydrate-bound complex structures. The two molecules in the asymmetric unit have similar overall characteristics (rmsd of 0.73 Å for all atoms) with little differences observed in some previously disordered regions. At the N-termini in particular molecule B presented some residual amino acids from the histidine-tag cleavage (Fig. 1) forming a tail lodging itself between two crystal-related symmetry molecules. Compared with the available structure, residues 1–3 were well ordered in both chains as well as Pro10 and Glu11. The CRD itself remains unchanged despite a slight movement in the adjacent loop composed of Arg74 and Gly75. This is in analogy to the recent observation that galectin-3 CRD remains largely unchanged upon ligand binding [31]. This solvent accessible region is stabilized thanks to hydrogen bonds and salt bridges with the surrounding residues (Glu58, Glu72) and with a symmetry-related molecule in molecule A only, while an extensive network of water molecules is present at the ligand binding site. Six residues showed potential alternate side chain conformation, namely Val18, Leu89, Asp95, Arg110, Arg117 and Arg133. None of these residues is known to be involved in ligand binding.

Molecule A had an unusual difference electron density around the buried thiol group (Cys38), which is not conserved among galectins (Fig. S1). Cysteine was replaced with an S-oxy cysteine in the structure, with potential oxidation due to radiation damage. This solvent accessible cysteine residue is not known to be involved in any biological function.

### 2-*O*-Benzylphosphate galactoside inhibitor 6 binding to hGal-7

**Fig. 2:**
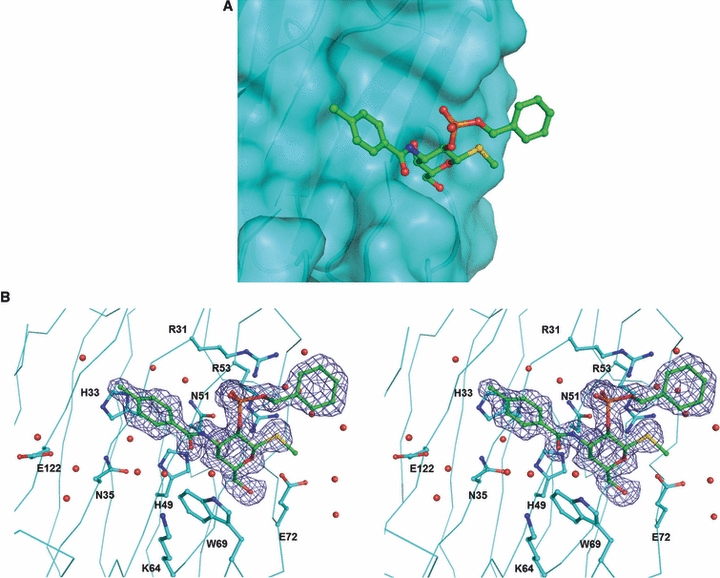
Structure of hGal-7 in complex with compound **6**. (A) hGal-7 surface (cyan) in complex with compound **6** (green). (B) hGal-7 (cyan) in complex with compound **6** (green). Stereo figure with electron density showing compound **6** (at 1σ level), residues involved in binding highlighted in stick and ball and water molecules in red spheres.

**Fig. 3:**
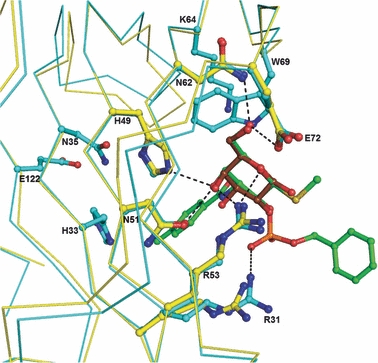
Comparison with galactose binding to hGal-7. hGal-7 in complex with compound **6** (cyan, green respectively) superposed with hGal-7 in complex with galactose (PDB code 2GAL, yellow). Residues involved in ligand interaction are highlighted in stick and ball.

The native protein does not show any major conformational change upon binding to the inhibitor **6** with an rmsd of 0.9 Å (all atoms, Fig. 1). The complex structure was solved to 1.7 Å (*R*_work_ = 20.4%, *R*_free_ *=* 23.9%, with 98.5% Ramachandran favoured) and electron density was clearly visible for the small molecule inhibitor in molecule B (Fig. 2), while only partial density could be seen in molecule A. The galactose moiety shows similar binding property to that of the galactose monomer (PDB 2GAL, Fig. 3) including the six main potential hydrogen interactions (Table 3). However, the high resolution structure highlights stronger binding achieved through the side groups of the 2-*O*-benzylphosphate inhibitor **6**. The phosphate group has a weak hydrogen bonding potential with Arg31, while it is also stabilized by hydrogen-bonded water molecules linked to the same Arg31 and Asn51. The amido group also shows potential interactions with water molecules linked to Lys64 and Trp69, broadening the binding capacity of the ligand to a region not previously involved for galactoside recognition by galectin-7. The phenyl group itself does not seem to be involved in the binding of ligand **6** despite being in close proximity to polar residues His33, Glu122 and Asn35. Interestingly, His33 and Glu122 are not conserved among galectins, thus focusing the ligand interaction towards this position could offer better specificity of inhibition. The anomeric sulfur atom can only weakly interact with a water molecule (3.5 Å) stabilized by Arg31. Interestingly, the benzyl moiety of the *O*-benzylphosphate **6** is not taking part in the inhibitor binding and faces away from the protein surface. The absence of interaction of the *O*-benzyl group is also reflected by the similar, or slightly better, affinity of the corresponding methyl phosphate **5**.

**Table 3:** Hydrogen bond interactions between hGal-7, compound 6 and β-galactose (PDB: 2GAL)

hGal-7	Compound **6**	Distance (Å)	β-galactose (PDB: 2GAL)	Distance (Å)
Asn51 OD1	O4A	3.0	O4A	3.2
His49 NE2	O4A	2.8	O4A	2.9
Arg53 NH2	O4A	3.0	O4A	3.2
Arg53 NH2	O5A	3.1	O5A	3.1
Glu72 OE2	O6A	2.8	O6A	2.5
Asn62 ND2	O6A	2.8	O6A	2.9
Arg31 NH1	O2P	3.4		
Water	O1P	2.9		
Water	O2P	2.7		
Water	O1B	2.7		
Water	O1B	3.1		
Arg74 N(sym)	O3P	2.8		
Arg71 NH1(sym)	O2P	2.6		

Furthermore, the difference in the crystal packing of the ligand complex structure, highlighted by the different crystallographic cell dimensions compared with the native structure, may be explained by the ligand interaction with a symmetry-related molecule involving residues Arg71 and Arg74 through two potential hydrogen bonds with the phosphate group (Table 3, Fig. S2). This symmetry-related molecule is unique to molecule B of the asymmetric unit and may also explain why only partial electron density was visible for the ligand of molecule A.

### Structural implications of inhibitor binding to hGal-7

Determination of the crystal structure of hGal-7 in complex with compound **6** supports our understanding of the binding affinity data obtained with other synthetic monosaccharide-based inhibitors (Table 1). Compounds **5** and **6** showed the best affinity for hGal-7 with a similar *K*_d_ range (240 and 450 μm respectively). These two molecules differ by the presence of the *O*-benzylphosphate group in **6** compared with an *O*-methylphosphate group in **5**. The *O*-benzylphosphate was shown not to be involved in the binding; however, the smaller methyl group might be able to interact with Arg31, possibly via a different orientation than that of the *O*-benzyl group, and hence show a slightly better affinity. The design of inhibitors with modified (alkyl)-phosphate groups, e.g. at the 2-O position, could be envisioned for a more favourable interaction with Arg31 and other nearby residues.

Talose-based compounds structurally related to **2**–**6** have recently been described as inhibitors of other galectins, including a taloside **7** (Table 1) that presents the *O*-benzylphosphate group in a position different from that of *O*-benzylphosphate **6** [32]. Inhibitors **6** and **7** differ at two more positions, in addition to the inverted stereochemistry at the pyranose C2: the amide group and anomeric sulfur atom. The first is likely to weaken the interactions with surrounding water molecules, whereas substitution of S for O should not perturb the weak solvent-mediated contact with Arg31. The taloside **7** has similar affinity for galectin-7, which suggests that inversion of the pyranose C2 results in presentation of the 2-*O*-benzylphosphate in a similarly favourable position.

Overall comparison with compound **2** demonstrates that addition of a 2-*O*-phosphate moiety dramatically improves the affinity of the galactoside inhibitors (*K*_d_ ≪ 1 mm). The presence of smaller (alkyl) substituents at the phosphate group provides possible interaction partners with residues from the hGal-7 CRD. Furthermore, the phenyl ring of the C3-benzamido group was revealed not to be involved in the ligand binding. This position on the inhibitor may, however, have potential for improved galectin selectivity by targeting polar residues unique to hGal-7 away from the CRD.

## Discussion

Galectin-7 is an important member of the galactose-binding family implicated in many pathological events. The availability of detailed structural information on the protein binding mechanism to its natural glycoconjugates has led to the development of synthetic inhibitors with enhanced affinity.

The high resolution structure of hGal-7 in complex with the 2-*O*-benzylphosphate galactoside inhibitor **6** shows that the synthetic molecule has retained all the conserved interactions involving the galactose moiety, from which it is derived, as well as offering new interactions. In particular, the phosphate group at the galactose O-2 position is involved in additional hydrogen and water mediated bonds with Arg31 and Asn51 of the CRD. Investigation of additional substituents at this position should lead to stronger inhibitors, considering the flexibility of these residues and the adjacent residue Arg53. The benzyl group appears to have no interaction with the protein and is orienting itself away from the CRD. This is to some degree analogous to the binding of disaccharides to galectins and was also observed with a galectin-1 inhibitor [16] where the second carbohydrate moiety faces away from the protein surface. The glycosidic sulfur atom brings limited power to the binding through its water-bonded sulfur atom; substitutions of this group may create better interaction with the protein, similar to earlier observations with substituted phenyl thio-galactosides [28].

On the other hand, the amido group of **6** is stabilizing the other side of the ligand by water-mediated interaction allowing the phenyl group to extend across the CRD. This position seems underexploited as galectin-7 presents a cluster of polar residues in a region showing a strong solvent network in the apoprotein. Further investigation to replace the benzamido group might help improve the inhibitor's affinity and selectivity toward galectin-7, since this region of the protein is not particularly conserved. A potent inhibitor of galectin-3 [33] carrying a 2,3,5,6-tetrafluoro-4-methoxybenzamido group at C3′ of lacNAc for example presented a stronger affinity due to an arginine–arene stacking and cation–π interaction involving the C3′-benzamido arene moiety.

The present structural study has highlighted the potential of a novel 2-*O*-benzylphosphate galactoside binding to hGal-7 (which is > 60-fold more potent than its parent galactoside) and gives a template for the design of stronger and selective galectin inhibitors. Availability of such molecules is essential to further refine our knowledge of lectin–ligand binding and to understand these proteins’ function and involvement in severe pathologies for which potent drugs are required.

## Experimental procedures

### Cloning, expression and purification of hGal-7

The gene coding for hGal-7 was obtained from RZPD (#IRAKp961G19141Q2, Berlin, Germany) and cloned into a modified pET-22 vector (Novagen, Darmstadt, Germany) with a cleavable N-terminal poly-histidine tag. BL21 (DE3) *Escherichia coli* cells were transformed with the recombinant plasmid. Cells were grown at 37 °C to an *A*_600_ of 0.6–0.7, and then 0.5 mm isopropyl thio-β-d-galactoside induction at 18 °C was allowed for expression of the recombinant protein. Cells were harvested 18 h after induction by centrifugation (4 °C, 6 500 ***g***). Induction of the protein was analysed by SDS/PAGE.

Cell pellets were resuspended in lysis buffer (50 mm sodium phosphate pH 8.0, 300 mm NaCl, 20 mm imidazole, 1 mm phenylmethanesulfonyl fluoride, 1 mg·mL^−1^ lysozyme), incubated on ice for 10 min and sonicated for 10 min by 30 s cycles. The preparation was then centrifuged for 30 min at 4 °C, 10 000 ***g***. The supernatant was loaded on a 5 mL HisTrap FF column (GE Healthcare, Uppsala, Sweden) pre-equilibrated with wash buffer (50 mm sodium phosphate pH 8.0, 300 mm NaCl, 20 mm imidazole). The column was washed extensively and a single step elution (50 mm sodium phosphate pH 8.0, 300 mm NaCl, 250 mm imidazole) was carried out. The eluted fraction was dialysed overnight in wash buffer at 4 °C. The N-terminal histidine tag was removed by HRV3C (Novagen, Darmstadt, Germany) cleavage for 18 h at 4 °C and the reaction was loaded onto a 1 mL HisTrap FF column (GE Healthcare, Uppsala, Sweden). The unbound fraction was collected and concentrated (Amicon Ultra; Millipore, Cork, Ireland) before loading on a gel filtration column (Superdex 200; GE Healthcare, Uppsala, Sweden) pre-equilibrated with final buffer (50 mm Tris pH 8.0, 200 mm NaCl). A single peak elution was analysed by SDS/PAGE and showed a band of pure hGal-7 at 15 kDa.

### Inhibitors and galectin-7 affinities

Synthesis of compounds **2–6** [29] and **7** [32] have been described in detail earlier and evaluation as galectin-7 inhibitors was performed as earlier reported [29].

### Crystallization

Crystals of recombinant hGal-7 were grown by the hanging-drop method, the protein at 10 mg·mL^−1^ in 50 mm Tris pH 8.0, 200 mm NaCl, against a reservoir solution containing 50 mm sodium phosphate pH 8.0, 300 mm NaCl, 20 mm imidazole and 17% poly(ethylene glycol) 3350. Single crystals appeared after 2 days at 16 °C. A complete data set to 1.4 Å was collected from one crystal in space group P2_1_2_1_2_1_, with unit cell dimensions *a* = 54.30, *b* = 65.11, *c* = 70.83 Å, and two hGal-7 molecules per asymmetric unit.

hGal-7 was incubated with 2 mm compound **6** for 2 h at room temperature before being set up for crystallization. Cocrystals of the complex were obtained by the hanging-drop method against 100 mm Bistris-propane pH 8.5, 200 mm sodium formate and 20% poly(ethylene glycol) 3350. Single crystals appeared after 24 h at 16 °C. A complete data set to 1.7 Å was collected from one crystal in space group P2_1_2_1_2_1_, with unit cell dimensions *a* = 35.3 Å, *b* = 53.5 Å, *c* = 138.6 Å, and two hGal-7 molecules per asymmetric unit.

### Data collection and structure determination

Data were collected at the Diamond Light Source (Oxford, UK), beamlines IO3 and IO4 which were equipped with a Quantum-4 CCD detector (Area Detector Systems Corporation, Poway, California, USA). Crystals were soaked in 25% poly(ethylene glycol) 3350 as cryoprotectant prior to data collection. The data were processed using hkl2000 [34] (Table 2).

Initial phases were obtained by the molecular replacement method using phaser [35,36] with the coordinates of hGal-7 (PDB: 1BKZ). Crystallographic refinement was carried out using refmac5 version 5.5 [35,37], and shelxp version 97-3 [38] for anisotropic refinement of the high resolution hGal-7 structure. Model fitting was done using coot version 0.6 [39]. The program molprobity [40] was used to check for validation of the structure. Detailed statistics for the refined structure of hGal-7 and its complex are given in Table 2. Figures were drawn with pymol (The PyMOL Molecular Graphics System, Version 1.3, Schrödinger, LLC, New York, NY, USA).

## Abbreviations

CRD: carbohydrate recognition domain
hGal-7: human galectin-7

## Supporting information

The following supplementary material is available:

**Fig. S1.** Structure of high resolutionhGal-7–Cys38. Positive electron density at Cys38 moleculeA.

**Fig. S2.** Structure of hGal-7 in complex withcompound **6**. Crystallographic symmetry interfaceinteracting with compound **6** and involving residues Arg71 and Arg74 through two potential hydrogen bonds with the phosphate group.

Please note: As a service to our authors and readers, this journal provides supporting information supplied by the authors. Such materials are peer-reviewed and may be re-organized for online delivery, but are not copy-edited or typeset. Technical support issues arising from supporting information (other than missing files) should be addressed to the authors.
