# Supplementary material for: Inhibition mechanism of human galectin-7 by a novel galactose-benzylphosphate inhibitor
Source: FEBS J. 2012 Jan;279(2):193–202. doi: 10.1111/j.1742-4658.2011.08414.x (PMC3328751; doi:10.1111/j.1742-4658.2011.08414.x)

the FEBS Journal
www.febsjournal.org

Inhibition mechanism of human galectin-7 by a novel galactose-benzylphosphate inhibitor

Geoffrey Masuyer, Talat Jabeen, Christopher T. Öberg, Hakon Leffler, Ulf J. Nilsson and K. Ravi Acharya

DOI: 10.1111/j.1742-4658.2011.08414.x

Supplementary material

Inhibition mechanism of human galectin-7 by a novel galactose-benzylphosphate inhibitor

Geoffrey Masuyer[1], Talat Jabeen[1], Christopher T. Öberg[2], Hakon Leffler[3], Ulf J. Nilsson[2] and K. Ravi Acharya[1]

Figure S1. Structure of high resolution hGal-7 – Cysteine 38. Positive electron density at the thiol group of Cysteine 38 molecule A.

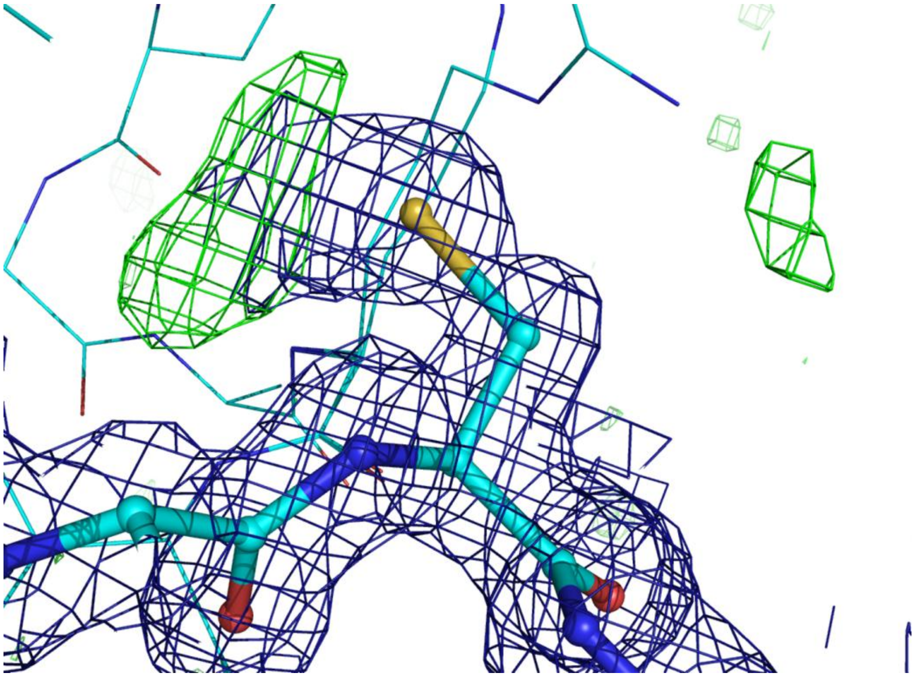

Figure S2. Structure of hGal-7 in complex with compound 6. Crystallographic symmetry interface interacting with compound **6** and involving residues Arg 71 and Arg 74 through two potential hydrogen bonds with the phosphate group

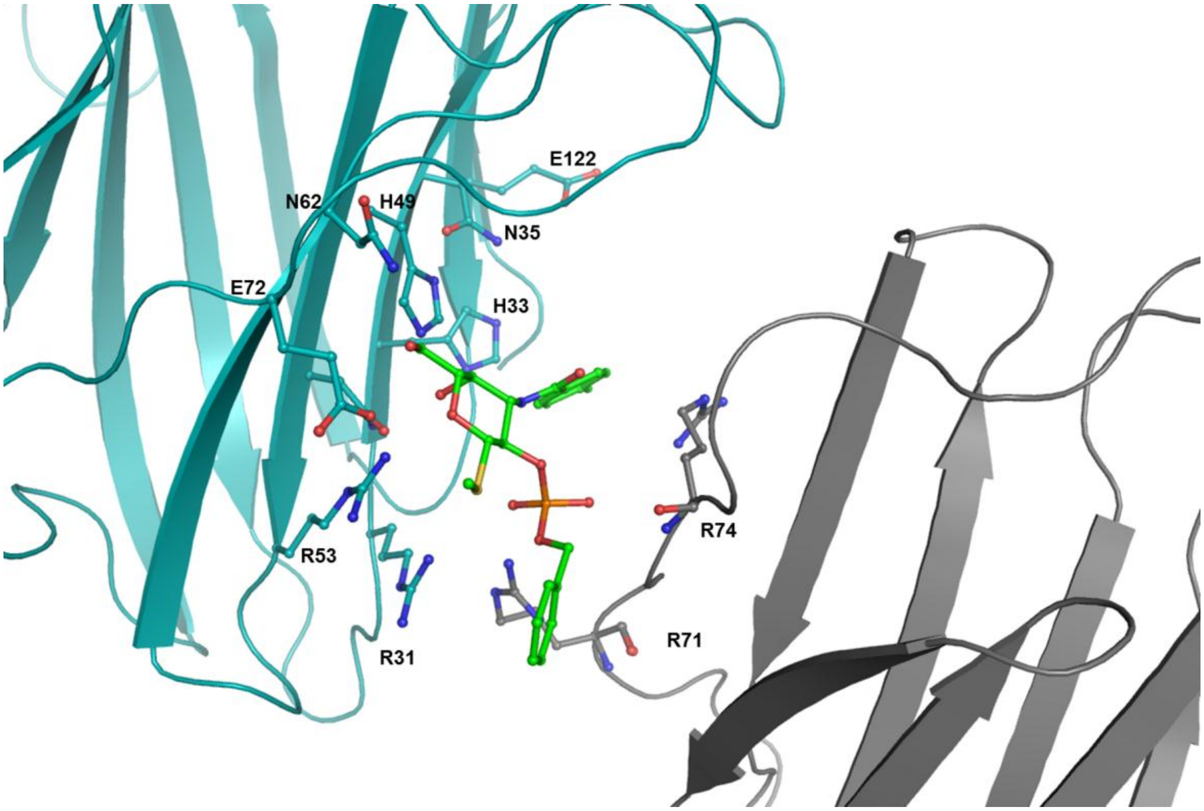

Supplement: Supplementary file 1 [file febs0279-0193-SD1.pdf]
